# Cases of Bronchocele

**Published:** 1822-10

**Authors:** J. B. Austin


ART. IV.—*Cases of Bronchocele.* By J. B. AUSTIN, Esq.

NOTICING the communication, p. 182 of your last Number, on the efficacy of Iodine in cases of Bronchocele, and the observation of your correspondent, that it is a disease very prevalent in Sussex; I beg to state that it is equally so in this elevated*

part of Surrey, and the adjoining county of Hants; and that patients afflicted with it have been in the constant practice of using the lozenges of burnt sponge, and in many cases, even of long standing, with much success.

I lately had two patients brought to me, sisters,—the elder about fourteen, the younger between eleven and twelve years of age; the tumors of both had been gradually increasing from childhood, and were now very prominent. In the elder, both lobes of the gland were nearly equally enlarged; the right lobe of the younger was the part most affected; in both, they were hard and unyielding to pressure. Wishing to try the comparative efficacy of iodine and the sponge lozenges, I furnished the elder with a concentrated tincture of iodine, and desired her to take ten drops twice a-day in water, and to increase it gradually to twenty drops. The younger I supplied with the lozenges of burnt sponge, as prepared by Shepherd, of Fleet-street, directing her to allow one gradually to dissolve under the tongue, night and morning; and I was assured of the perseverance of both by the superintendance of a careful mother. The elder girl bore an increase of the tincture to fifteen or sixteen drops without inconvenience, but on reaching twenty she was obliged to desist awhile, on account of sickness, vertigo, and some disturbance of the bowels; all which, however, soon went off without the assistance of medicine, and after about a week she recommenced upon a dose of twelve drops, which she increased to fifteen, eighteen, and eventually to twenty, without much disorder, though occasionally obliged to desist a day or two. After having taken it about three weeks, the tumor was softer and its measure less, and at the expiration of two months it had nearly disappeared. She was desired to persevere another month; and now not any remains of it are perceptible, and her health is excellent.—The younger sister took the lozenges daily, and in less than a month her tumor was evidently less, but afterwards it did not decrease so rapidly as that of her elder sister; and even now, three months from her commencing the lozenges, there is still left a little tumefaction, and a small indurated portion about the size of a hazel-nut.

I have been in the habit of recommending the sponge lozenges very confidently, from observing their efficacy, if not in entirely removing the tumors, at least in rendering them much softer, and materially lessening them; and in most cases, when bronchocele *first* appears in young females, the use of them for a fortnight only will cause its total disappearance. The cases related above were of some years' standing, and within the last

---

* Haslemere is nearly upon a level with Hind Head, which is stated to be about 930 feet above the level of the sea.

three had increased very rapidly. As our locality offers many opportunities of observation, I purpose giving the tinct. iodine a very fair trial: should the results be favourable, I may probably trouble you with them, if worth your notice, on some future occasion.

*Haslemere; August 6th,* 1822.

